# Behavioral spillover between the use of reusable shopping bags and recycling at home: A field experiment

**DOI:** 10.1371/journal.pone.0328259

**Published:** 2025-08-11

**Authors:** Claudia Arias, Carlos A. Trujillo

**Affiliations:** 1 School of Business, CESA, Bogotá, Colombia; 2 School of Management, Universidad de los Andes, Bogotá, Colombia; Guangxi Normal University, CHINA

## Abstract

This study examines positive behavioral spillover between waste-related behaviors that vary in complexity. Specifically, we test whether encouraging a relatively simple behavior—using reusable shopping bags—can lead to increased adoption of a more complex behavior, such as recycling. To investigate this, we conducted a longitudinal field experiment. Additionally, we explored the moderating roles of three individual-level constructs: Perceived Difficulty, Self-efficacy, and Perceived Consumer Effectiveness. Our findings show that positive spillover occurs for recycling at home, but not for recycling elsewhere. Moreover, Perceived Difficulty strengthens this effect, while Perceived Consumer Effectiveness diminishes it. We conclude by discussing how these insights can inform public and private interventions aimed at promoting recycling.

## Introduction

Waste generation has escalated over the past decades due to population growth and consumerist lifestyles, contributing significantly to air and water pollution, land degradation, and greenhouse gas emissions. Waste generation has dramatically increased in recent years, with a global increase of 73% in the volume of municipal solid waste projected between 2020 and 2050, from 2.01 to 3.40 billion tonnes [1,2]. Therefore, solid waste has become an acute environmental problem [2]. Among the strategies to mitigate this crisis, recycling remains a central pro-environmental behavior promoted by governments and organizations worldwide, and it is one of the targets of sustainable consumption by 2030 [3]. Recycling contributes to reducing the amount of waste that would otherwise be disposed of in landfills, seas, and streets; it also helps to conserve natural resources [4], improves the quality of the living environment [5,6], and thereby contributes to environmental sustainability, understood as the responsible interaction with the environment to avoid depletion or degradation of natural resources and allow for long-term environmental quality [7–12].

Yet, despite its acknowledged importance, recycling adoption remains limited across diverse populations—only 14% of global waste is recycled [1]. A persistent challenge has been the gap between pro-environmental attitudes and actual behavior, commonly referred to as the “green gap” [13,14]. Thus, identifying new mechanisms to translate intention into action is imperative [15] and this challenge has been the subject of nearly fifty years of research.

One promising mechanism to address this issue is behavioral spillover—the idea that performing one pro-environmental behavior may influence the likelihood of performing another [16,17]. Research on positive spillovers is particularly relevant in the context of waste-related behaviors for several reasons. First, waste management comprises a suite of interconnected actions, including reduction, reuse, and recycling [18]. Nevertheless, the current literature has devoted more effort to examining these practices in isolation [19], which limits our understanding of how engagement in one may activate another. Second, although multiple factors have been studied in relation to recycling, limited attention has been given to the role of other related behaviors [e.g., reducing and reusing] in facilitating recycling through spillover effects. Third, prior studies have predominantly examined the spillover from recycling to other behaviors [20–22], overlooking the reverse direction [23,24].

Most prior research has treated spillovers as incidental outcomes rather than intentional tools for behavior change [25–27]. Thus, the literature explains behavioral spillover as the effect that an initial, targeted pro-environmental behavior [PEB 1], encouraged by an intervention [e.g., policy, campaign, or incentive], has on a subsequent, unintended pro-environmental behavior [PEB 2] [16,17,28]. In contrast, our study explores spillover not merely as a side effect but as a deliberate strategy to promote a target behavior. Using the theory of behavioral spillover, we propose a different model of intervention to promote the adoption of a specific behavior—recycling—as the target subsequent behavior [PEB 2].

The literature suggests that the occurrence of predictable spillover is facilitated by two key features of the incumbent behaviors: connection to an overarching common goal [17,29] and differences in behavioral difficulty, with difficulty being an important factor influencing the adoption of any behavior [30–33]. Although the literature has suggested that these two conditions likely favor positive spillovers [17,28], to the best of our knowledge, we are proposing a novel way to model and test an intervention that combines both features. Specifically, we designed an intervention based on message framing to examine whether encouraging a relatively easy reusing behavior [using reusable shopping bags, PEB 1] can lead to the adoption of a more complex one [recycling, PEB 2]. Despite the relevance of this pathway, few studies have explored whether an easier reducing/reusing behavior can deliberately foster recycling [23,34].

Our research directly addresses this gap. The relationship between simpler actions—such as reusing shopping bags—and more complex behaviors—like recycling—has practical significance for policymakers and practitioners who design environmental interventions. Simpler behaviors may act as entry points into sustainable lifestyles, building momentum and strengthening a pro-environmental identity that supports continued engagement.

In addition to studying the spillover phenomenon, prior research has highlighted the importance of identifying the conditions and mechanisms that facilitate this effect. Previous studies on spillover and recycling have examined mediators such as pride and environmental identity [21,35,36], environmental concern [37,38], and the goal of waste reduction [29]. Fewer efforts have focused on moderators. Among these, some studies have explored the role of intervention type [39], anticipated guilt [37,40], and consideration of future consequences. Thus, there is limited understanding of how individual perceptions influence this process.

We therefore test the influence of three psychological constructs as moderators of the spillover effect between reusing and recycling: Perceived Difficulty, Self-Efficacy, and Perceived Consumer Effectiveness [PCE]. In doing so, our study integrates insights from the Theory of Planned Behavior [30], Social Cognitive Theory [41], and sustainability research on spillover and consumer beliefs [27,28]. Although some research has involved these variables in spillover studies [21,42], we aim to reinforce the role of individual beliefs in positive spillovers and contribute a novel element by including perceived consumer effectiveness as a moderator in this context.

Our contribution is twofold. First, we extend the emerging literature on strategic behavioral spillovers by demonstrating that interventions can deliberately target easier behaviors to trigger more challenging ones [24,43]. Studying the transition from reusing to recycling offers insights into how habits evolve and allows for the strategic use of behavioral spillover as a tool, rather than relying on it as a passive consequence. Second, we identify the boundary conditions under which such interventions are most effective, exploring why and when the spillover from simple to complex waste-related behaviors may occur. Together, our contributions have direct implications for the design of campaigns and policies aimed at promoting sustainable lifestyles. To put our ideas to the test, we conducted a field experiment in a real-world context. Thus, our study contributes to the growing body of research that uses experimental methodology to test spillover effects [25,34,36].

## Literature review

### Definition and approaches to behavioral spillovers

Behavioral spillover occurs when engagement in one behavior increases [or decreases] the likelihood of engaging in another related behavior [44,45].

Prior literature has identified both positive and negative effects. When individuals behave in a particular way, they may be more likely to adopt a subsequent related behavior—this is referred to as positive spillover [17,43,46]. Conversely, a behavior may also reduce the likelihood of future pro-environmental actions or even promote negative subsequent behaviors—this is known as negative spillover [47,48].

Although positive spillovers are often viewed as desirable—particularly in the sustainability domain due to their potential to amplify the effects of individual-level interventions across areas such as energy use, waste management, and sustainable consumption [25,38,43] —recent literature advises caution against overly optimistic interpretations. Critics highlight that spillovers are often weak, inconsistent, and highly context-dependent [16,46,49]. Additional studies point to factors such as perceived effort, insufficient infrastructure, and the nature of the initial behavior as contributors to rebound effects or behavioral inertia [50]. Moreover, empirical evidence on the directionality of spillovers remains mixed. While some research shows that actions like waste sorting or setting personal energy-saving goals can promote further green behaviors [51], other studies reveal no effect—or even negative spillovers— depending on the subsequent behavior [52].

Most of the existing research has conceptualized spillover as an incidental or unintended side effect of interventions—possibly explaining why both positive and negative spillovers are observed. However, a growing body of work suggests that spillovers can be deliberately induced [53,54], opening the door to strategic interventions aimed at scaling pro-environmental behavior. These studies advocate for the intentional use of spillovers as a viable strategy to drive behavioral change [43,53], especially in contexts like sustainability, where behavioral adoption often unfolds in chains or clusters [48,49]. Our study embraces this latter approach, building on the concept of positive spillover as a strategic tool, and contributes to the limited body of research that has tested deliberate spillovers from easy to complex behaviors [43].

### Behavioral similarity and complexity as features to positive spillovers

As stated previously, behavioral spillover is a phenomenon that may be useful in promoting sustainable behaviors [40,43,51,53,55]. Positive effects found in prior research encourage the need for more targeted and deliberate approaches. Before designing an intervention for this purpose, it is important to acknowledge that the occurrence of spillover is influenced by characteristics of the incumbent behaviors as perceived by the individual, such as desire for consistency, environmental self-identity, and behavioral similarity [17,28]. In simple terms, behaviors affected by spillover must be related in some way [24]. In our intervention, we involve one type of relatedness: similarity around a common goal.

Categorizing behaviors around a common goal is particularly useful in the sustainability domain, where individuals’ pro-environmental behaviors can be aligned with a broader principle or objective [e.g., behaviors that contribute to improved waste management] [17]. The achievement of such a goal can be an important motivation for engaging in related behaviors [see 41]. Following this reasoning, we focus our research on reducing/reusing and recycling behaviors, both of which are aligned with the shared goal of reducing solid waste. This should facilitate positive spillovers between them. Additionally, behaviors may differ in certain characteristics, such as behavioral difficulty or complexity, which makes some pro-environmental behaviors easier to adopt than others. This study takes advantage of this distinction to design an intervention that favors the adoption of recycling.

Recycling is more complex than reusing in several ways. Recycling involves:

a] multiple interrelated actions [e.g., using different bags, cleaning materials, and taking them to the correct separation container], b] understanding complex information [e.g., types of materials, eco-labels], and c] managing expected outcomes [e.g., separating waste appropriately and disposing of it according to the available infrastructure to complete the recycling cycle].

These elements increase its objective complexity [33,56]. Furthermore, evaluating whether a city has adequate infrastructure adds contextual complexity [57]. Therefore, people may subjectively perceive recycling as a difficult task [33,58,59]. In contrast, using a reusable bag simply involves purchasing the bag and taking it to the supermarket, which makes it a much easier behavior.

Previous research has suggested that people tend to prefer simpler behaviors [60] and that spillover may follow a “foot-in-the-door” logic [61], whereby initial, easier behaviors pave the way for the adoption of more demanding ones [24,43]. Few studies have investigated positive spillovers from easy to complex behaviors [see 43]. Our work contributes to filling this gap.

By combining both features—goal similarity and differences in behavioral difficulty—we propose that promoting an easy behavior [reusing] could trigger a more complex behavior [recycling], especially when the behaviors are perceived as sequential and goal-congruent. Specifically, our reasoning is that recycling is inherently more complex than using a reusable shopping bag, and when perceived as part of a behavioral sequence, this contrast may catalyze upward spillover between them [24,43]. Based on this, we present our first hypothesis:

H1. The use of reusable shopping bags increases the adoption of recycling behavior [positive spillover].

### Moderating individual-level factors

Our second objective is to explore moderating factors that facilitate the occurrence of spillover. As previously explained, similarity between behaviors, the use of comparable resources, and orientation toward the same goal are essential elements that establish connections between behaviors and increase the likelihood of positive spillovers [17,28]. In addition, previous literature highlights the relevance of environmental knowledge and identification with sustainability for establishing behavioral similarity and promoting positive spillovers [17]. However, although behavioral similarity and difficulty set the conditions for positive spillovers, not all individuals respond in the same way. Therefore, we examine three individual-level moderators that may shape the spillover process: perceived difficulty, self-efficacy, and perceived consumer effectiveness [PCE].

#### Perceived difficulty.

Perceived difficulty reflects a person’s subjective assessment of how challenging a behavior is [62]. Previous research suggests that perceived difficulty affects not only the adoption of individual behaviors but also how individuals link one behavior to another [32,63]. People tend to favor simpler behaviors and may avoid complex ones if they are perceived as too effortful [60]. If recycling is perceived as especially difficult, the motivational effect of a preceding behavior [e.g., using a reusable bag] may not be enough to induce further action. Therefore, we expect perceived difficulty to moderate the spillover from reusing to recycling as follows:

H2. The spillover between reusing and recycling behaviors is stronger [vs. weaker] for people with lower [vs. higher] levels of perceived difficulty about recycling.

#### Self-efficacy.

Self-efficacy refers to an individual’s belief in their capacity to perform a behavior [41,64]. It is a well-established predictor of pro-environmental behaviors such as energy saving and recycling [65,66], and may also influence spillover effects [see 38,54,67]. Moreover, self-efficacy has been identified as a moderator in other relationships, such as those between attitudes and behavior [68]. Previous research suggests that individuals with high self-efficacy are more likely to transfer behaviors across domains because their confidence helps them overcome perceived challenges [43]. Hence, we propose the following:

H3. The spillover between reusing and recycling behaviors is stronger [vs. weaker] among people with high [vs. low] levels of self-efficacy to recycle.

#### Perceived consumer effectiveness [PCE].

Perceived Consumer Effectiveness [PCE] refers to the belief that one’s actions can contribute meaningfully to environmental outcomes [69,70]. While PCE has been widely studied as a direct antecedent of sustainable behaviors [27,64,66,71], its role as a moderator of spillover effects remains unexplored. We suggest that individuals with high PCE may not depend on sequential behavioral cues, as they already feel empowered to act sustainably. In contrast, individuals with low PCE may benefit more from a behavioral chain in which a simple action prompts a more complex one [72]. These individuals may rely on behavioral continuity to reinforce the perceived effectiveness of their actions. Thus, we hypothesize:

H4. The spillover between reusing and recycling behaviors is more salient [vs. less salient] among people with weaker [vs. stronger] environmental PCE.

To further refine our analysis, we distinguish between general PCE and specific PCE. While general PCE refers to broad environmental impacts, specific PCE refers to the perceived effectiveness of specific actions in achieving targeted environmental goals [70]. Given that our study focuses on a targeted behavior—recycling—we complement the general PCE analysis by also examining the moderating effect of specific PCE related to recycling. Thus, we propose:

H5. The spillover between reusing and recycling behaviors is more salient [vs. less salient] among people with weaker [vs. stronger] specific PCE [i.e., the belief that recycling can achieve waste management goals].

## Materials and methods

Most research on spillovers uses cross-sectional data and correlational designs to estimate potential relationships among pro-environmental behaviors [23,73]. Some research has used experimental or longitudinal studies to test causal relationships over time [25,34,36]. This approach is useful to truly test the spillover phenomenon when promoting targeted sustainable behaviors [53]. Thus, through a longitudinal experimental design, we test weather promoting the use of reusable shopping bags [easy behavior] influence the target, more complex behavior of recycling.

**Fig 1:**
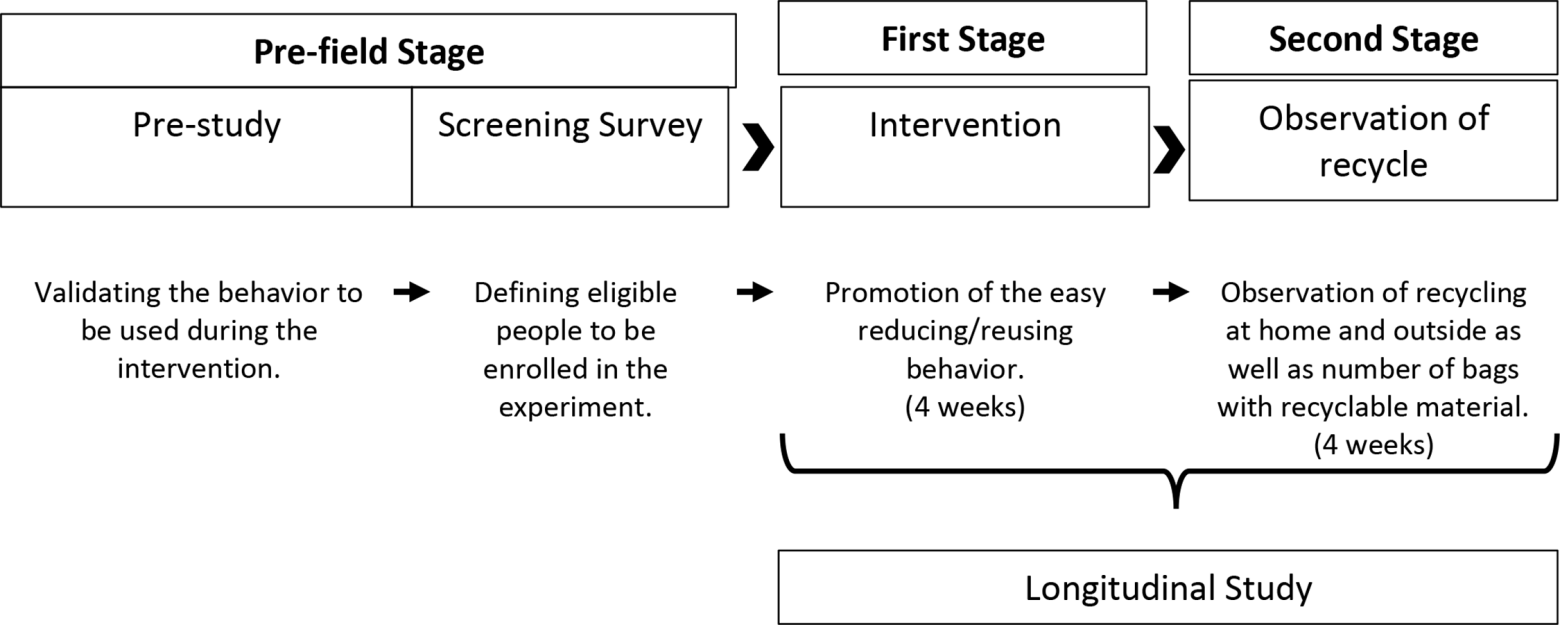
Stages involved in the field experiment.

We conducted a field experiment in a real context where actual behaviors occur. In Fig 1 we show the three stages of the field experiment.

### Pre-field stage

This phase consists of a pre-study with the following purposes: 1] to validate the differences in perceived complexity of the reducing/reusing and recycling behaviors, 2] to identify a phrase that captures a common goal for both behaviors.

First, we validated the perceived complexity of reusing and recycling. We conducted a survey with 167 individuals from a local university, including students, faculty members, and administrative staff. They shared similar characteristics to the sample we later recruited for the field experiment. We asked participants about perceived difficulty [How difficult is it for you? in a scale from 1 = Very easy to 7 = Very difficult] and frequency [How often do you? in a scale from Never = 1 to Always = 5] of different behaviors including reducing/reusing and recycling ones. A t-test confirmed that reusing was perceived as significantly easier than recycling [Mrecycle at home = 2.94; Mrecycle outside=3.31; Mreusable bags = 2.43; t_recycle at home – reusable bags = −3.55; p = 0.00; t_recycle outside – reusable bags = −5.36; p = 0.00].

Second, we needed to determine a way to state a common goal for reusing and recycling. To do that, we asked participants in the same survey to select a phrase that they thought mostly captured the potential contributions of reusing and recycling to sustainability from a drop-down list that included a set of phrases worded as goals. These goals came from prior research about the objectives of reducing, reusing, and recycling [74,75] [see Table 1 for a full list of goals]. Participants associated recycling [25%] and using reusable shopping bags [33%] with the goal of ‘Reducing waste and litter that end up in landfills, streets, the sea, and other places’. Also, we identified that 71% of people ranked this goal among the top three according to its importance. Thus, this phrase was the common goal that aligned reducing/reusing behavior with recycling, making them similar and therefore prone to spillover effects [47].

**Table 1:** Set of goals assessed [goals related to reducing, reusing, and recycling behaviors].

Goals	References
Reducing waste and litter that end up in landfills, streets, the sea, and other places	[74,75]
Reducing materials	
Conserving natural resources and saving the environment	
Helping my community and other communities	
Increasing beauty of nature and enhancing an esthetic experience	
Promoting better health and quality of life, avoiding sickness [less germs, bugs…]	
Providing long-term well-being for future generations	

Additionally, this phase involved participant screening and recruiting: to find the sample of the experiment we screened and selected participants using two criteria: a] people who attribute high levels of importance to the phrase/goal that aligns reducing/reusing and recycling behaviors, to increase the likelihood of engagement during the intervention, b] people who do not already recycle or use reusable bags very frequently, to avoid ceiling effects in either the intervention or the dependent variable of the experiment.

To recruit participants, we sent an online call to participate in a paid lifestyle related research to students, faculty members, and administrative staff from two business schools in Bogota, Colombia. The invitation described the main characteristics of the field study: duration, payment [USD 24, which is 10% of the minimum wage of Colombia, approximately]. This quantity – COP$ 80,000 – is calculated based on the average exchange rate corresponding to July-October of 2019 [the period in which people participated in the field study], and was split in two payments: 50% at the beginning and 50% at the end of the study], and format [online, so participants could answer questionnaires and report activities from any place and with any electronic device]. From 221 people who signed up for the study, 191 answered a screening questionnaire that specifically asked about the goal’s level of importance and the frequency of adoption of the behaviors of interest. According to the criteria previously mentioned, only 65 people were eligible for participation in the study. That is, people who rated the importance of the goal with 5 or above in a 1–7 scale and do not frequently use reusable bags or recycle. The recruitment period for this study started on 27/05/2019 and ended on 16/06/2019.

All procedures involving human participants were reviewed and approved by the Ethics Committee of the School of Management at Universidad de Los Andes [General Memorandum No. 50]. The study was conducted in accordance with the ethical principles of the Declaration of Helsinki and applicable national regulations concerning confidentiality, informed consent, and voluntary participation. Participants were fully informed about the purpose of the study and the nature of their involvement, and were assured that their responses would remain confidential and anonymous. Written informed consent was obtained from all participants prior to their involvement in the study.

### First phase: Intervention

This phase consisted of a four-week intervention period in which we manipulated the use of reusable shopping bags via a message-based prompt. Participants were randomly assigned to one group [32 to the treatment group and 33 to the control group]. In the treatment group we encouraged reducing/reusing behavior while the control group would not receive any treatment.

At the beginning of the four weeks, both groups received an email that directed them to answer a survey that included the variables of interest [i.e., perceived difficulty, self-efficacy, perceived consumer effectiveness, reducing/reusing and recycling behaviors] as well as psychographic covariates. We henceforth will call this test 1. The survey included the participants’ consent to the field experiment [see the specific participants’ consent in Supplementary Material]. Once participants responded survey, they received the instructions to report their behaviors during the next four weeks on a website called Lifestyle Diary. This website was created specifically to the purpose of the study [see the layout in S1 Appendix. In the e-mail we asked both groups to report habits and behaviors on four categories [i.e., entertainment, shopping, sports and sustainability]. We involved other categories besides sustainability to avoid social desirability bias [76,77].

The intervention consisted of a message-based prompt to incorporate a new behavior—bringing reusable bags for shopping—into participants’ daily routine. This was framed as an action contributing to a shared environmental goal [“reducing waste and litter that end up in landfills, streets, the sea, and other places”], previously identified by participants as personally relevant. Participants in the treatment group received weekly reminders to report this activity in a dedicated section [“New Activities”] of their Lifestyle Diary.

Although minimal in nature, this type of message-based intervention is theoretically grounded in the literature on behavioral spillovers [17,43]. Prior research has shown that even subtle prompts or framed messages can activate self-perceptions or environmental goals, leading to the adoption of additional sustainable behaviors [78,79]. Thus, this behavioral cue—combined with goal alignment and sustained weekly engagement—constitutes a valid and theory-informed manipulation of the independent variable.

Every week, treatment and control groups received a reminder by email, with a link to the questionnaire in the Lifestyle Diary. When participants accessed their Lifestyle Diary, they found a section for each of the four weeks that directed them to the corresponding questionnaire. After completing the four weeks, they received an email that thanked them for their participation in the first phase of the study. Additionally, they were directed to a questionnaire to advance to the next phase. This survey was a retake of test 1. We will call this test 2. It included the same variables to observe changes after the four weeks. People who did not answer test 2 were dropped from the study and received only the initial 50% of the payment for their participation in the first phase of the study.

#### Instruments and variables for phase 1.

Table 2 shows the questions used in test 1 and test 2. Both tests asked for frequency of reducing/reusing, recycling, main constructs, and control variables. These covariates came from previous research on determinants of recycling behavior. We included moral and social norms, environmental attitudes and identity, as well as knowledge [13,80]. Table 2 also shows the items and scales we used and adapted to operationalize each variable, as well as Cronbach’s alphas, which shows that we achieved acceptable reliability for all scales.

**Table 2:** Operationalization of variables.

Variable	Authors	Items	Scale	Cronbach’s
**Independent**				
Use of reusable shopping bags	[81]	How often do you carry reusable bags for shopping?’ [t1-t2] ‘In the last week, when you could, how often did you use reusable shopping bags?’[intervention]	1:Never- 5:Always	
**Dependent**				
Recycling behavior		**How often do you:** - Identify the colors of the bins that represent each recyclable material -Know the materials that correspond to each bin -Clean and condition recyclable material before sorting -Take recyclable material to the collection point -Recycle [separating garbage] at home - Recycle [separating garbage] outside the home	1:Never- 5:Always	Test 1: 0.88 Test 2: 0.89
**Moderators**				
Perceived difficulty	[62]	**How difficult is it for you?** - To identify the colors of the bins that represent each recyclable material -To know the materials that correspond to each bin -To clean and condition recyclable material before sorting -To take recyclable material to the collection point -To recycle [separating garbage] at home - To recycle [separating garbage] outside the home	1: Very easy – 7: Very difficult	Test 1: 0.89 Test 2: 0.85
Self-efficacy	[82,83]	**If requested, how confident are you that you can?** - Identify the colors of the bins that represent each recyclable material -Know the materials that correspond to each bin -Clean and condition recyclable material before sorting -Take recyclable material to the collection point -Recycle [separating garbage] at home - Recycle [separating garbage] outside the home	1: Not confident I can at all – 7: Highly confident I can	Test 1: 0.77 Test 2: 0.88
Perceived Consumer Effectiveness [Environmental]	[69]	**As individual, I feel through my actions I can contribute to:** - Solving environmental problems - Solving loss of biodiversity [flora and fauna] - Avoiding pollution and indiscriminate use of water **-** Avoiding climate change - Solving waste issues	1: Disagree – 4: Agree	Test 1: 0.74 Test 2: 0.72
Perceived Consumer Effectiveness of recycling to achieve the waste-related goal		As individual, I feel that by carrying reusable bags for shopping, I can contribute to reducing waste and litter that end up in landfills, streets, the sea, and other places. As individual, I feel that by recycling, I can contribute to reducing waste and litter that end up in landfills, streets, the sea, and other places.	1: Disagree – 4: Agree	
**Control**				
Environmental Concern	[69]	- Environmental problems are not affecting my life personally. - Environmental problems are exaggerated because in the long-run things balance out. -I have too many obligations to take an active part in an environmental organization. - I can think of many things I’d rather do than work toward improving the environment.	1: Disagree – 4: Agree	
Moral Norm [recycling]	[84]	-I feel a strong personal obligation to recycle -I am willing to put an extra effort into recycle -I would feel guilty if I do not recycle	1: Disagree – 4: Agree	Test 1: 0.61 Test 2: 0.74
Social Norm [recycling]	[85]	-People who are important to me expect me to recycle -My neighbors and friends expect me to recycle	1: Disagree – 4: Agree	Test 1: 0.71 Test 2: 0.64
Environmental Identity	[79]	‘I think of myself as an environmentally conscious person’ ‘I think of myself as a loss of biodiversity conscious person’, ‘I think of myself as a pollution and indiscriminate use of water conscious person’ ‘I think of myself as a climate change conscious person’ ‘I think of myself as a waste issues conscious person’	1: Disagree – 4: Agree	Test 1:0.77 Test 2: 0.84
Attitude towards recycling	[81]	For you, recycling [separate garbage from recyclable materials] is:	Good-Bad Inappropriate – Appropriate	Test 1: 0.86 Test 2: 0.93
Perceived Knowledge [recycling]	Scale proposed based on the definition of perceived knowledge of [86]	-I know how recycling works at my place of residence [building/condominium] - I know the conditions [shape, cleaning] to sort recyclable material - I know how to separate organic from recyclable material - I know the potentially recyclable materials	1: Disagree – 4: Agree	Test 1: 0.80 Test 2: 0.81
Sociodemographics		Gender Age Marital Status Level of Education Income Occupation Housing type Household size [# people; area in sqm]		

### Second phase: Longitudinal observation of recycling

This phase consisted of another four-week longitudinal record of self-reported activities right after the four-week intervention. Once participants answered test 2 as the final step of the intervention phase, we asked participants in both groups to undertake two new activities as part of their everyday routine and report them in a new Lifestyle Dairy during the next four weeks. To cover the real purpose of this phase [observing recycling] we told participants that they could choose one activity, and that the other activity would be assigned by the experimenter randomly. Therefore, participants could choose one activity in any of the four categories we already explained: entertainment, shopping, sports, and sustainability. The second activity was the same for all participants: recycling. To report it, participants answered different types of questions: 1] Self-reported frequency, corresponding to the observed behavior: “During the last week, how often did you separate organic from recyclable material at home?; “During the last week, how often did you separate organic from recyclable material outside?” on a five-point scale [1 = never – 5 = always]. 2] We asked participants to report the number of bags containing only recyclable material they produced during the last week.

Every week, both treatment and control groups received a reminder by email to report their behaviors using a link to the Lifestyle Diary. Once again, the Lifestyle Diary had a dedicated section for each week [weeks 5, 6, 7 and 8] that guided participants to the questionnaire report. At the end of the eight weeks, participants received an email that thanked them for their participation in the study during both phases and instruction for final payment of the second 50%. Note that this method improves the reliability of self-report by following some of the recommendations of [87]: 1] The use of a repeated report using a diary 2] The simplification of the report trough guidance and training and 3] Minimize the retrospective task by asking every week.

## Results

### Descriptive statistics and correlation analyses

The final number of participants, after a few people withdrew between phases 1 and 2, was 58 [31 = treatment group; 27 = control group], for a total number of observations of 232, which constitutes the final sample size. Participants were 55% women. Age ranged from 18 to 57 [Mage = 29 years; SD = 7.76]. Most participants were employed [67.2%], with a monthly household income between $305 USD - $1.524 USD [COP$ 1,000,000–5,000,000] [Mincome = $1.802USD; SD = $1.922]. Household size ranged between 30 and 500 sqm [Marea = 102 sqm; SD = 82.5 sqm]. Average number of people living together: [Mpeople = 3 persons; SD = 1.62]. Table 3 summarizes the descriptive statistics of the sample.

**Table 3:** Descriptive statistics of the sample.

Sociodemographics	Percentage
**Age**	
[18–25]	36.2
[26–35]	51.8
[36–45]	6.9
[46–55]	3.4
[56 or over]	1.7
**Gender**	
Male	44.8
Female	55.2
**Occupation**	
Employee	67.3
Student	17.2
Freelance	8.6
Other	6.9
**Level of Education**	
High school	27.6
Undergraduate	36.2
Postgraduate diploma	25.9
Master	10.3
**Household Income**	
<COP$ 1,000,000	14.0
COP$ 1,000,000–3,000,000	28.1
COP$ 3,000,001–5,000,000	24.6
COP$ 5,000,001–10,000,000	19.3
> COP$ 10,000,000	14.0
**Housing Type**	
Single-family house	39.7
Building [apartment complex]	46.5
House complex	8.6
Other	5.2
**Household Size [people]**	
1–2 people	34.5
3–5 people	50.0
> 5 people	12.1
NR	3.4
**Household Size [area]**	
< 50 sqm	10.3
50–100 sqm	55.2
101–150 sqm	8.6
151–200 sqm	10.3
> 200 sqm	5.2
NR	10.3

During the observation stage, we obtained 4 observations per participant, hence the final sample was 232 data points. On average, participants in the treatment group occasionally used reusable shopping bags during the four weeks of intervention [M = 3.35; SD 1.07]. People in the treatment group displayed a significant positive change [from t1, M = 3.42 to t2, M = 3.71] in this behavior compared to those in the control group [t1, M = 3.74 to t2, M = 3.42]; [Mtreatment = 0.29; SD = 0.93; Mcontrol = −0.26; SD = 0.89; Wilcoxon signed-rank test: z = −2.39; p = 0.017]. We used this non-parametric test because of differences in variance between the two groups and the relatively small number of participants in each group. Regarding recycling behavior, participants reported that, over four weeks, they often recycled at home [M = 3.84; SD = 0.94] and outside [M = 3.67; SD = 0.93]. We recalculated measurements of recycling to adjust for household size and number of people. We did this because these are two factors that influence waste volume and recycling frequency. Thus, we calculated frequency of recycling and number of bags, per person and per square meter [sqm]. Table 4 contains the full descriptive results of recycling. After the intervention, during the 4 weeks of observation, frequency of recycling per person was significantly higher in the treatment group than in the control group [Mtreatment = 1.60; SD = 0.88; Mcontrol = 1.36; SD = 0.84; Wilcoxon signed-rank test: z = −2.20; p = 0.028] suggesting that promoting reusable bags during the 4 weeks of intervention period generated a spillover towards recycling at home in the following 4 weeks.

**Table 4:** Means and correlations among recycling behaviors.

Variables [Recycling decisions]	Mean [SD]	1.	2.	3.	4.	5.	6.
1. Treatment	3.35 [1.07]	___					
Frequency of recycling at home	3.84 [0.94]						
2. Frequency of recycling at home *[per person]*		.15**	___				
3. Frequency of recycling at home *[per sqm]*		.05	.63***	___			
Number of bags with recyclable material	77.6% [up to 3 bags]						
4. Number of bags with recyclable material *[per person]*		−.04	.35***	.24***	___		
5. Number of bags with recyclable material *[per sqm]*		−0.3	.23***	.55***	−.80***	___	
6. Frequency of recycling outside home	3.67 [0.93]	−0.5	.28***	.16**	.18***	.15**	___

N = 232 observations

Note: Correlation including treatment was calculated through point-biserial correlation. Significance level: ** p < 0.05; *** p < 0.01

Regarding the potentially moderating constructs that we measured, we evaluate if there were changes before and after the intervention [test 1 and test 2]. We found the following: Before [test 1] Perceived difficulty of recycling [Mpdr = 3.35; SD = 1.41]; Self-efficacy [Mser = 5.41; SD = 1.09], environmental PCE [Mpce_e = 3.54; SD = 0.47] and recycling PCE [Mrpce = 3.57;SD = 0.75] [see Table 5]. After the intervention [test 2] we found: perceived difficulty of recycling [Mpdr = 3.09; SD = 1.25]; self-efficacy [Mser = 5.61; SD = 1.11]; environmental PCE [Mpce_e = 3.67; SD = 0.38] and recycling PCE [Mrpce = 3.65; SD = 0.48] [see Table 6]. There were not significant differences between test 1 and test 2 for perceived difficulty of recycling and self-efficacy [Pr[binomial]pdr = 0.90; Pr[binomial]ser = 0.24] which shows that the intervention did not affect potentially these moderating constructs. Conversely, there were significant differences between test 1 and test 2 for environmental and recycling PCE [Pr[binomial]pce_e = 0.02; Pr[binomial]rpce = 0.00]. Also, we calculated correlations between these constructs and found that before and after the intervention, the relationship among constructs remained stable. We also found that PCEs of using reusable shopping bags and recycling were positively correlated, which is consistent with the alignment of reducing/reusing and recycling behaviors towards the same goal. Also, the measures of recycling were positively associated, suggesting an underlying behavioral pattern including frequency, quantity, and behaviors inside/outside home.

**Table 5:** Descriptive statistics of independent and moderator variables before the intervention [test 1].

Variables [t1]	Mean [SD]	1.	2.	3.	4.	5.	6.
1. Treatment	3.35 [1.07]	___					
2. Perceived Difficulty	3.35 [1.41]	.05	___				
3. Self-efficacy	5.41 [1.09]	−.02	−.53***	___			
4. General PCE	3.54 [.47]	.11	−.29***	.17***	___		
5. Specific PCE [Recycling] *Recycling on environmental goal*	3.57 [.75]	.15**	−.07	.17**	.60***	___	
6. Specific PCE [Bags] *Use reusable shopping bags on environmental goal*	3.65 [.69]	−.15**	.03	.13**	.04	.10	___

N = 232 observations

Note: Correlation including treatment was calculated through point-biserial correlation. Significance level: ** p < 0.05; *** p < 0.01

**Table 6:** Descriptive statistics of independent and moderating variables after the intervention [test 2].

Variables [t2]	Mean [SD]	1.	2.	3.	4.	5.	6.
1. Treatment	3.35 [1.07]	___					
2. Perceived Difficulty	3.09 [1.25]	.05	___				
3. Self-efficacy	5.61 [1.11]	.02	−.73***	___			
4. General PCE	3.67 [.38]	.05	−.12	.16**	___		
5. Specific PCE [Recycling] *Recycling on environmental goal*	3.65 [.48]	−.02	−.28***	.28***	.37***	___	
6. Specific PCE [Bags] *Use reusable shopping bags on environmental goal*	3.77 [.49]	−.09	−.10	.24***	.33***	.36***	___

N = 232 observations

Note: Correlation including treatment was calculated through point-biserial correlation. Significance level: ** p < 0.05; *** p < 0.01

### Behavioral spillover between reducing/reusing and recycling

We performed two manipulation checks using again a Wilcoxon signed-rank test. The first check confirmed significant differences in perceived difficulty [z = −4.27; p = 0.00]. Recycling was perceived as more difficult than using reusable shopping bags. The second manipulation check assessed whether the intervention in the treatment group successfully increased the use of reusable bags. For this check, we estimated the change in the use of reusable shopping bags from before and after the four intervention weeks. Results revealed a significant difference between treatment and control groups [z = −2.39; p = 0.017]. There was a positive change in the reducing/reusing behavior in the treatment group [13% on average] whereas there was a decrease in the control group. [see Table 7].

**Table 7:** Use of reusable shopping bags by condition.

Group	Variable	Mean	SD	[95% Conf.Interval]	
Treatment	Use of reusable bags [t1]	3.42	1.06	3.03	3.81
	Use of reusable bags [t2]	3.71	1.22	3.26	4.15
Control	Use of reusable bags [t1]	3.74	1.23	3.25	4.23
	Use of reusable bags [t2]	3.48	1.12	3.04	3.93

#### Hypotheses tests.

To test H1 [*The use of reusable shopping bags increases the adoption of recycling behavior]* we conducted several linear regressions for panel data. The analysis examined the effect of treatment [dummy coded treatment = 1; control = 0] on recycling behaviors during the second stage of the observations [weeks 5–8]. Hence, we had 4 observations in the dependent variables per participant, one per each week. We estimated five multiple regression models in which the dependent variables were each recycling decisions, namely: 1] frequency of recycling outside, 2] frequency of recycling at home per person, 3] per sqm, 4] the number of bags filled with recyclable material per person and 5] per sqm. We found significant effects for frequency of recycling at home, normalized per person or sqm. As Table 8 shows, we found a positive and significant effect of treatment on the frequency of recycling at home per person [Coeff = 1.57; p = 0.00], which supports the first hypothesis [H1]. Treatment had no significant effects on the outcome variables of recycling outside the home and the number of bags filled with recyclable material [per person and per sqm]. We will later discuss the meaning and implications of finding effects for frequency of recycling at home, but not outside, and not in the volume of recycled material. S2 Appendix shows these regression models. Given this sample size [232 observations], we conducted a post-hoc power analysis. Assuming a middle size effect [Cohen`s f = 0.15] alpha = 0.05 and 12 predictors, we obtained a power of 0.98, which means that the risk of type 1 o 2 errors in our analyses given our sample size was very small. To further guarantee that our results are reliable despite a relatively small panel, we replicated regressions for recycling at home per person and per sqm using Generalized Estimating Equations [GEE] procedure with clustered errors [88]. The results are consistent. S3 Appendix contains the full results of these regressions.

**Table 8:** Regression model for recycling behavior [frequency at home].

Variables	Recycling [Frequency at home]							
	*Per person*				*Per sqm*			
	B	SE	z	p	B	SE	z	p
Constant/Intercept	10.24	4.31	2.38	.01	−1.97	.30	−.66	.51
Treatment [vs. control] *[Use of reusable bags]*	1.57	.40	3.91	**.00**	.015	.033	.46	.65
***Variables of interest***								
Perceived Difficulty [recycling]	−.68	.26	−2.63	**.00**	−.004	.019	−.19	.85
Self-efficacy [recycling]	−.15	.19	−.81	.42	−.003	.015	−.20	.84
General PCE	−.35	.40	−.89	.37	−.006	.028	−.21	.83
Specific PCE [recycling]	1.02	.41	2.52	**.01**	.037	.034	1.08	.28
***Control variables***								
Environmental Concern	−.12	.06	−1.84	.07	−.001	.005	−.24	.81
Moral Norm [recycling]	.27	.27	−.99	.32	−.003	.019	−.18	.86
Social Norm [recycling]	.16	.21	.73	.47	−.018	.017	−1.06	.29
Environmental Identity	−2.56	.61	−4.17	**.00**	−.045	.060	−.76	.45
Knowledge [recycling]	1.97	.48	4.12	**.00**	.056	.042	1.32	.19
Attitude [recycling]	.31	.60	.52	.60	.074	.043	1.74	.08
**Gender**								
*Male*	1.05	.41	2.56	**.01**	.010	.039	.26	.80
Age	−.07	.03	−2.36	**.02**	.002	.002	1.03	.30
**Marital Status**								
*Married*	.42	.40	1.05	.30	−.004	.029	−.14	.89
*Living Together*	.55	.65	.85	.40	.011	.047	.23	.82
*Divorced*	2.67	1.29	2.04	**.04**	−.095	.095	−1.00	.32
**Level of Education**								
*Undergraduate*	2.23	.70	3.20	**.00**	.00	.06	.01	.99
*Graduate [post-graduate]*	.49	.47	1.04	.30	.02	.03	.55	.58
*Graduate [master]*	1.64	.70	2.35	**.02**	−.01	.05	−.18	.86
**Occupation**								
*Student*	1.36	.59	2.29	**.02**	.022	.045	.50	.62
*Independent job*	.13	.56	.23	.82	−.004	.040	−.10	.92
*Other*	−.91	.71	−1.28	.20	−.042	.054	−.77	.44
Income	−.03	.14	−.22	.82	.002	.010	.21	.84
**Type of housing**								
*Building [apartment complex]*	.26	.36	.72	.47	−.030	.026	−1.15	.25
*Houses complex*	.09	.68	.15	.88	−.088	.048	−1.83	.07
*Other*	−1.90	.87	−2.20	**.03**	−.017	.059	−0.29	.77
**Household size**								
*# People in the home*					−.012	.010	−1.18	.24
*Area [sqm]*	−.00	.00	−2.61	**.00**				

*Note: These categorical variables were dummy coded with the following reference category: Gender [Female]; Marital Status [Single]; Level of Education [High School]; Occupation [Employee]; Type of housing [Single-family home].

To test the robustness of these results we approached the analysis from a different perspective. We tested the individual level recycling trajectory over the course of the observation period combining data from stage 1 and stage 2 of the study. To do this, we created a new variable that captured changes in the recycling frequency at home at the individual level. Such “trajectory” variable was calculated in the following way: *Trajectory* *=* *post [after 4 weeks] recycling frequency at home – pre [beginning of the study] recycling frequency at home* *+* *average recycling frequency at home per person during the second stage 4 [weeks 5–8].* This way, we could test the effect of our treatment on these individual trajectories. We then conducted a linear regression on this new variable, with a dummy variable for treatment and controlling for age, gender, education and income. We again found a significant effect of being in the treatment [Coeff = 0.64; t = 2.94; p = 0.00], confirming the previous result.

Then we tested the hypotheses about moderating effects of perceived difficulty, self-efficacy and PCEs.:

- The spillover between reusing and recycling behaviors is stronger [vs weaker] for people with lower [vs higher] levels of perceived difficulty about recycling [H2].
- The spillover between reusing and recycling behaviors is stronger among those with higher levels of self-efficacy to recycle [H3].
- The spillover between reusing and recycling behaviors is more salient among people with weaker Environmental PCE [H4].
- The spillover between reusing and recycling behaviors is more salient among people with weaker Specific PCE [such about recycling behavior to achieve a waste management goal] [H5].

**Fig 2:**
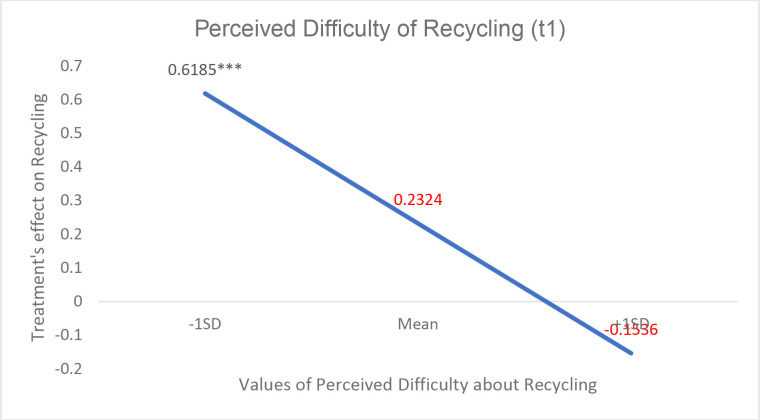
Treatment’s effect on recycling behavior [spillover effect] at different levels of perceived difficulty as a moderator. Values are estimated effect sizes. *** p < 0,001, ** p < 0,01 and * p < 0,05.

**Fig 3:**
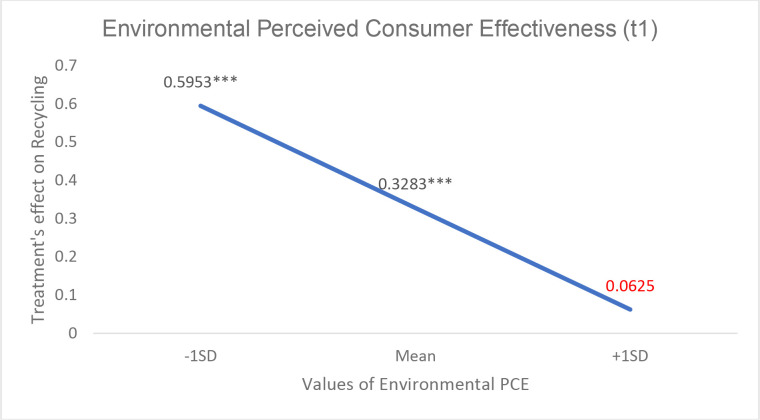
Treatment’s effect on recycling behavior [spillover effect] at different levels of Environmental PCE as a moderator. Values are estimated effect sizes. *** p < 0,001, ** p < 0,01 and * p < 0,05.

**Fig 4:**
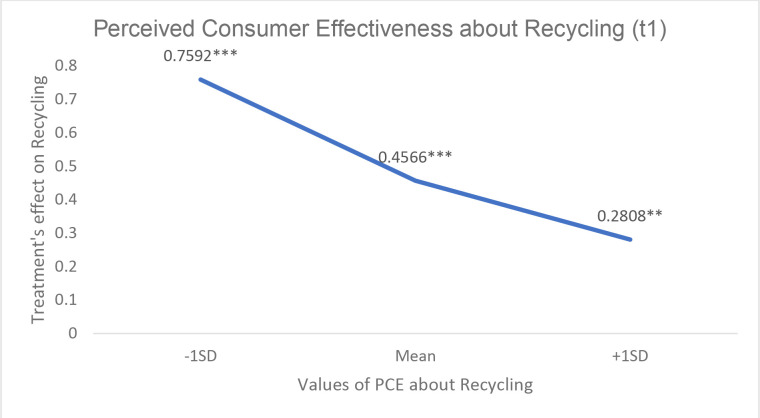
Treatment’s effect on recycling behavior [spillover effect] at different levels of Specific PCE [about recycling] as a moderator. Values are estimated effect sizes. *** p < 0,001, ** p < 0,01 and * p < 0,05.

We estimated moderation effects for each variable of interest using a linear regression approach. We used bias-corrected bootstrapping for 95% confidence intervals [CI] based on 5,000 bootstrap samples [using the plug-in PROCESS 3.4.1, Model 1 in SPSS] [89]. These regression models included interaction terms between treatment and each moderating variable. We also probed moderation at −1SD, mean and +1SD of the moderating variables. Table 9 shows moderating effects of perceived difficulty and PCE on the spillover between the use of reusable shopping bags and recycling behavior. Figs 2–4 also show these conditional effects. A significant interaction was found between perceived difficulty of recycling and treatment [Coeff_int = −0.27; F = 9.07; p = .003] affecting the magnitude of the spillover effect. This confirms H2. As the estimation of conditional effects shows, the spillover is significant for those who reported a relatively low Perceived Difficulty [−1SD; Coeff = .62; t = 3.58; p = .000] while the effect is not significant for those bracketed at average and above [+1SD]. Thus, being part of the treatment group increases the frequency of recycling per person by 0.62 SD when the perceived difficulty of recycling is relatively low [1.9 in a scale from 1 to 7]. H3 was not confirmed. The interaction between self-efficacy and treatment was **not** significant [Coeff_int = −0.12; F = 0.99; p = .321].

**Table 9:** Results of the moderation analysis.

Variables		Conditional Effects of the focal predictor at values of the moderators				
Moderators	Values	Effect [SE]	t	p values	Bootstrapped 95% Confidence Interval	
					Low Level	Upper Level
*Perceived Difficulty*						
−1SD [−1.417]	1.93	.62 [.17]	3.58	.000	.2776	.9594
Mean	3.35	.23 [.12]	1.86	.064	−.0136	.4785
+1SD [+1.417]	4.00 a	−.15 [.18]	−.83	.406	−.5180	.2107-
*Environmental PCE*						
−1SD [−.467]	3.08	.56 [.17]	3.20	.001	.2143	.9010
Mean	3.55	.32 [.13]	2.54	.011	.0722	.5733
+1SD [+.467]	4.00 a	.09 [.18]	.503	.615	−.2598	.4376
Specific PCE [about recycling] *						
−1SD [−.747]	2.82	.76 [.20]	3.75	.000	.3600	1.1584
Mean	3.57	.46 [.12]	3.68	.000	.2115	.7016
+1SD [+.747]	4.00 a	.28 [.14]	1.98	.048	.0017	.5600

Note: * Perceived consumer effectiveness about recycling behavior to achieve the goal of “Reducing waste and litter that end up in landfills, streets, the sea, and other places”.

a. One SD above the mean is above the maximum observed in the data for the moderator variable, so the maximum measurement for this variable in the scale is used for conditioning instead.

Regarding Perceived Consumer Effectiveness [PCE], we found a significant interaction with treatment [Coeff_int = −0.49; F = 3.75; p = .054] confirming H4. The interaction probing shows that the effect of treatment is positive and significant for people whose PCE is relatively low [−1SD] [Coeff = .60; p = .00]. As PCE increased, the effect decreases but it is still significant for people with average PCE. However, for people with relatively high PCE [+1SD]], being in the treatment group does not have any effect on recycling. We also found support for H4a. There is a significant interaction between PCE about recycling and the treatment group [Coeff_int = −0.39; F = 4.33; p = .038]. Although the effect is significant across all levels of PCE about recycling, the pattern is similar [see Fig 4]; as PCE about recycling increases, the effect of the treatment on recycling decreases.

## Discussion

In this study, using a longitudinal field experiment, we tested whether encouraging the adoption of a relatively easy behavior—using reusable shopping bags—through a message-based prompts intervention, could serve as a gateway to engaging in a more complex behavior, such as recycling. This was based on the well-documented theory of behavioral spillovers, which highlights similarity around a common goal and differences in difficulty as two key features that foster positive effects. We found evidence supporting this positive spillover from simple to complex waste-related behaviors. This finding aligns with recent literature emphasizing the importance of waste-related goals in enabling positive spillovers between complementary behaviors, which are essential for advancing toward a circular economy [29]. Our results extend the scope of this research by empirically confirming the spillover between circular behaviors through an experimental design.

However, we found the effect only for the frequency of recycling at home. There was no effect for recycling frequency outside the home or for the number of bags of recycled material. This suggests that the activation of recycling through the promotion of reusable bags leads people to recycle more frequently in spaces where they have volitional control. In contrast, the availability of infrastructure for recycling outside the home [e.g., separate bins] can interfere with what people are able to do. In addition, the lack of observed effects on the number of recycling bags may indicate that recycling volume was not affected. This is surprising because if people are recycling more frequently, we might expect an increase in the number of bags. However, since we do not know the weight of the bags, we may speculate that the increased frequency of recycling raised the weight of each bag rather than their number.

Our finding that spillover occurred primarily in the domestic context aligns with previous research emphasizing the importance of volitional space and contextual convenience in sustaining pro-environmental behaviors [36,49]. The absence of an effect on recycling outside the home contradicts prior findings suggesting broader behavioral transferability. For example, [39] found that tourists often carry over pro-environmental habits from home to travel settings, indicating some degree of cross-contextual behavioral consistency. However, our findings suggest a need to consider more recent research questioning the robustness of this transfer. [2], for instance, found that while intentions and behaviors were significantly linked in home settings, the same was not true for workplace recycling, indicating a contextual barrier to spillover. Similarly, [90] highlighted that organizational factors, such as logistical support and social norms, were stronger predictors of workplace recycling than personal attitudes formed at home. Moreover, value systems, social norms, and cultural frameworks play a central role in shaping pro-environmental behaviors, and may also influence how behavioral spillovers unfold across contexts. For instance, [91,92] show that in Chinese workplaces, low-carbon behaviors are driven not only by individual values but also by relational motivations, as employees adopt such behaviors to reinforce social ties [guanxi] and shared values. These mechanisms suggest that the pathways through which spillovers occur may vary significantly across cultures, depending on how pro-environmental actions are socially interpreted and motivated, and that external infrastructures or social norms may act as boundary conditions that limit the effectiveness of such spillover effects.

Additionally, although we controlled for numerous covariates, other variables—such as habit strength [93] or the availability of local recycling bins—may have influenced the results. Habit strength has consistently emerged as one of the strongest predictors of recycling engagement [94,95], and infrastructure, particularly the availability and accessibility of recycling facilities, plays a critical role. In fact, some studies have shown that recycling behavior only follows when infrastructure is present, and that organizational support with logistical infrastructure is a stronger predictor of recycling at work than personal environmental attitudes. This reinforces the idea that the physical context can constrain or enable recycling behavior—and any related spillover effect [90]. These factors should be tested or included in future research.

Our results support previous literature on the gradual shift from easy to difficult activities, which includes early studies on the ‘foot-in-the-door’ effect and others in pro-environmental contexts such as recycling, water conservation, and wind power [43,61,96,97]. Hence, we contribute to the still scarce research in the area of spill-overs from simple to complex behaviors [see 26,38,40,48]. In addition, our research could set a new path to encourage pro-environmental actions in the domain of waste-related behaviors.

Beyond confirming the existence of positive spillovers, our findings highlight a nuanced interplay between individual beliefs and the potential for behavioral transfer. The observation that spillover is more likely to occur under low perceived difficulty reinforces the idea that behaviors perceived as more manageable are more likely to catalyze further engagement] [30,43,60]. This aligns with the theoretical proposition that low perceived barriers can activate consistency-driven actions [17]. This highlights the potential for designing interventions that, given a target behavior such as recycling, simultaneously reduce perceptions of complexity, and promote other simpler related behaviors like the use of reusable bags.

Interestingly, PCE moderated the spillover such that individuals with lower perceptions of their behavioral impact were more influenced by the intervention. This finding suggests that spillover-based interventions may be particularly valuable for individuals who are less confident in the environmental impact of their actions, offering a complementary pathway to belief-based strategies [64,69,98]. In doing so, our research reinforces the importance of studying the interaction among different variables to better understand recycling and other pro-environmental behaviors [13,80]. For instance, future research may examine whether social connections [see 2,97] also moderate the strength of spillover effects, but in the opposite direction, that is, more social connections enhance the spillover. This may reveal novel dynamics of individual and social factors in their influence of pro-environmental behaviors.

Our findings did not support self-efficacy as a moderating factor in waste-related behavioral spillover [43,98], diverging from previous studies highlighting its central role. For example, [43] identified self-efficacy as a mediator that helps individuals progress from simple to complex environmental behaviors when they feel confident. This aligns with [21], which showed that self-efficacy partially mediates the spillover from low-carbon purchasing to recycling, particularly when environmental identity is involved. Conversely, [99] found that self-efficacy alone did not uniquely predict intentions for future behaviors compared to self-identity, implying its influence may be conditional. Similarly, [100] revealed limited evidence that attitude strength moderated spillover, raising questions about the situational boundaries of psychological drivers like self-efficacy, typically viewed as direct motivators of pro-environmental behavior. Despite self-efficacy not functioning as a moderator in our study, it may still serve as a meaningful mediator in conjunction with motivational and identity-based processes.

While most studies have viewed spillovers as incidental effects [26,27,101], we suggest that interventions can deliberately leverage positive spillovers to encourage specific behaviors, contributing to literature advocating this approach [53,54]. We aim to explore new intervention pathways by identifying similarities and differences between behaviors—like goal-related similarity and perceived difficulty—that facilitate positive spillovers. Our findings indicate that future research on pro-environmental behavior should analyze target behavior characteristics as a lens for guiding research and intervention design.

### Limitations and future research

Using a longitudinal field experiment in a real context where the behaviors occur, allowed us to develop a novel approach to promote the adoption of a specific behavior like recycling. This extends previous cross-sectional analyses [e.g., 21,26,41] and experimental studies [25,36] that tested positive relationships between behaviors. Additionally, the present work supports what previous research has highlighted about the importance of experimental studies, outside the lab, to obtain information about the practical relevance of spillover for pro-environmental behaviors [53]. Nonetheless, attrition during the 8 weeks of observation left the study with a relatively small sample size even though we achieved enough statistical power. We employed various econometric procedures to ensure the robustness of our results. Still, studies with larger, more varied samples are necessary to determine the precise scope of interventions in different populations. Although our sample included a diversity of individuals affiliated with the university setting—such as students, faculty, and administrative staff—it remains a relatively homogeneous group in terms of educational environment. As such, the findings of this study are not readily generalizable to broader populations beyond the university context. The specific characteristics of academic environments—such as greater access to information, institutional norms that promote sustainability, and peer influence—may have influenced participants’ responsiveness to the intervention, even though the observed effects were limited to behaviors performed at home. For instance, evidence suggests that individuals tend to adopt pro-environmental behaviors observed in close peers or colleagues to build or reinforce social bonds [91,92], a dynamic that may be particularly strong in university communities. Future research should test whether similar spillover effects emerge in non-academic or culturally distinct settings to better assess the external validity of our conclusions.

Another limitation is that we relied on self-reported observations. As explained, we mitigated some of the shortcomings of self-report by using a data collection method that increased accuracy by reducing the temporal gap between behavior and report, and by providing a context that facilitated the recording of responses. However, we did not directly observed behavior. Further studies may use direct observation. Finally, we use perceived difficulty, self-efficacy and PCE as moderators, but this is not an exhaustive list of potential moderators. There may other moderating variables that help explain how spillover occur. In addition, we assume linear moderating effects, but there could be more complex effects that are worth exploring to uncover in detailed the complex and multifaceted effects on recycling behavior. Further research should address this.

## Conclusions

### Managerial and policy implications

Campaigns about recycling and other sustainable behaviors focus mostly on increasing knowledge and awareness. Some appeal to make salient a sense of responsibility about waste and the consequences of incorrectly dispose it. Perhaps, all these efforts have been successful to increase a positive attitude about recycling; however, low rates of recycled material in different contexts show that people may be concerned and aware about recycling, but they do not actually take action [“the behavioral green gap” in 14,76]. To face this phenomenon, our research offers a novel perspective in which behavioral interventions could make use of action itself, encouraging and facilitating sustainable behaviors and practices, for example, to favor other practices like recycling. Thus, our findings offer actionable insights for policymakers, environmental organizations, and managers designing programs to foster recycling and other sustainable behaviors.

First, the evidence of positive behavioral spillover from reusable bag use to home recycling indicates that targeting relatively easy behaviors can serve as an effective entry point for more complex pro-environmental practices. Thus, interventions that begin with promoting low-barrier, goal-aligned behaviors, such as reducing or reusing, may catalyze broader engagement in sustainability efforts. Public campaigns could strategically prioritize easy-to-adopt habits—like using reusable shopping bags—not as end goals, but as behavioral gateways to encourage more challenging practices like consistent household recycling. As [23] suggested, public and private organizations may direct their efforts towards the adoption of easy behaviors that then will end up in other more complex ones.

Second, the fact that spillover effects occurred mainly in the domestic context underscores the importance of tailoring interventions to environments where individuals have greater volitional control. Local governments and community programs should focus on enabling home-based recycling through supportive policies, such as providing clearly labeled bins, regular waste collection, and feedback on recycling outcomes. These structural supports can complement behavior-based nudges and are crucial for sustaining behavior in the absence of formal regulation.

Moreover, the moderating role of perceived consumer effectiveness [PCE] suggests that communication strategies should be segmented. For individuals with high PCE, direct appeals to their impact may suffice. In contrast, for individuals with lower PCE, combining simple behavioral prompts with visible environmental benefits may be more effective. This segmentation could improve the efficiency of both public sustainability campaigns and corporate initiatives.

The findings also stress the importance of addressing perceptions of difficulty. Managers and policy designers should not only promote the benefits of recycling but also invest in reducing perceived complexity, for instance by offering instructional content, digital tools [e.g., apps to identify recyclable materials], or public signage that simplifies decision-making. These changes can lower psychological barriers and expand the reach of spillover effects.

Finally, the study’s null findings for self-efficacy as a moderator—but its acknowledged role as a mediator in other contexts—indicate that interventions should be multidimensional, combining individual beliefs around efficacy, effectiveness, and behavior-based strategies to optimize outcomes.

## Supporting information

S1 Appendix Layout of lifestyle diaries. (DOCX)

S2 Appendix Regression models [no significant effects]. (DOCX)

S3 Appendix GEE panel data regressions of recycling at home, per person, with interaction terms. (DOCX)
