# Supplementary material for: Behavioral spillover between the use of reusable shopping bags and recycling at home: A field experiment
Source: PLoS One. 2025 Aug 11;20(8):e0328259. doi: 10.1371/journal.pone.0328259 (PMC12338783; doi:10.1371/journal.pone.0328259)
Supplement: S1 Appendix — (DOCX) [file pone.0328259.s001.docx]

**S1 Appendix. Layout of lifestyle diaries.**


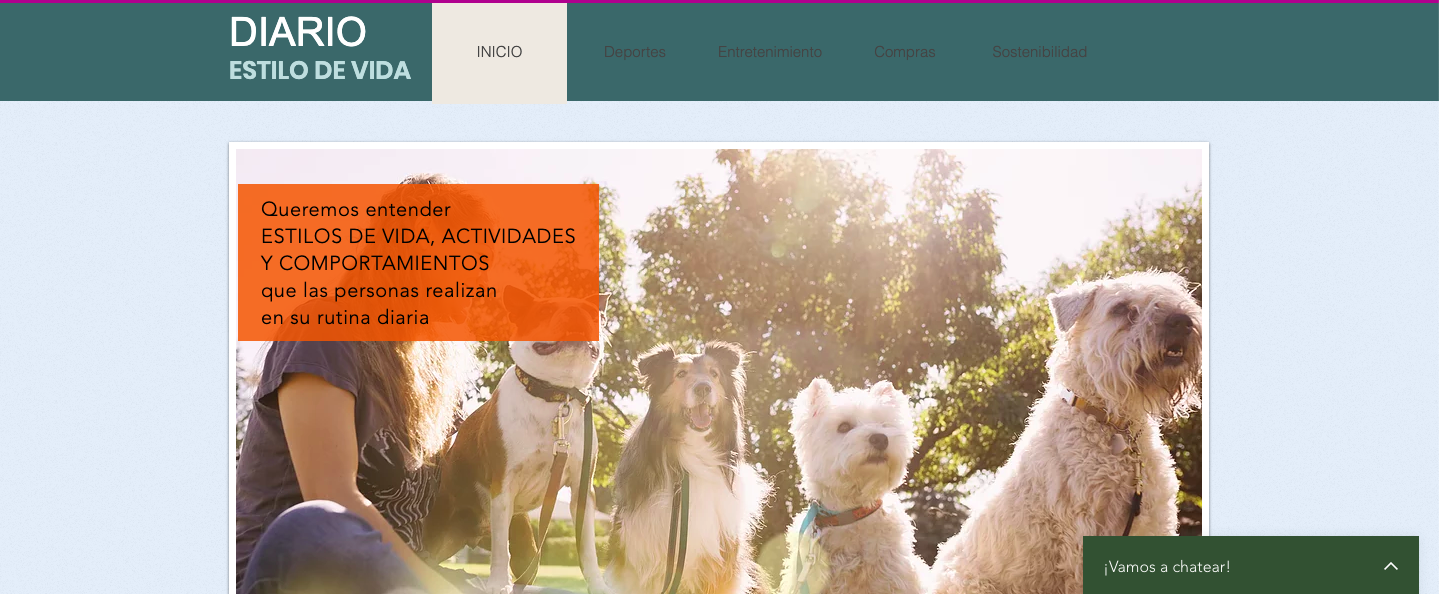

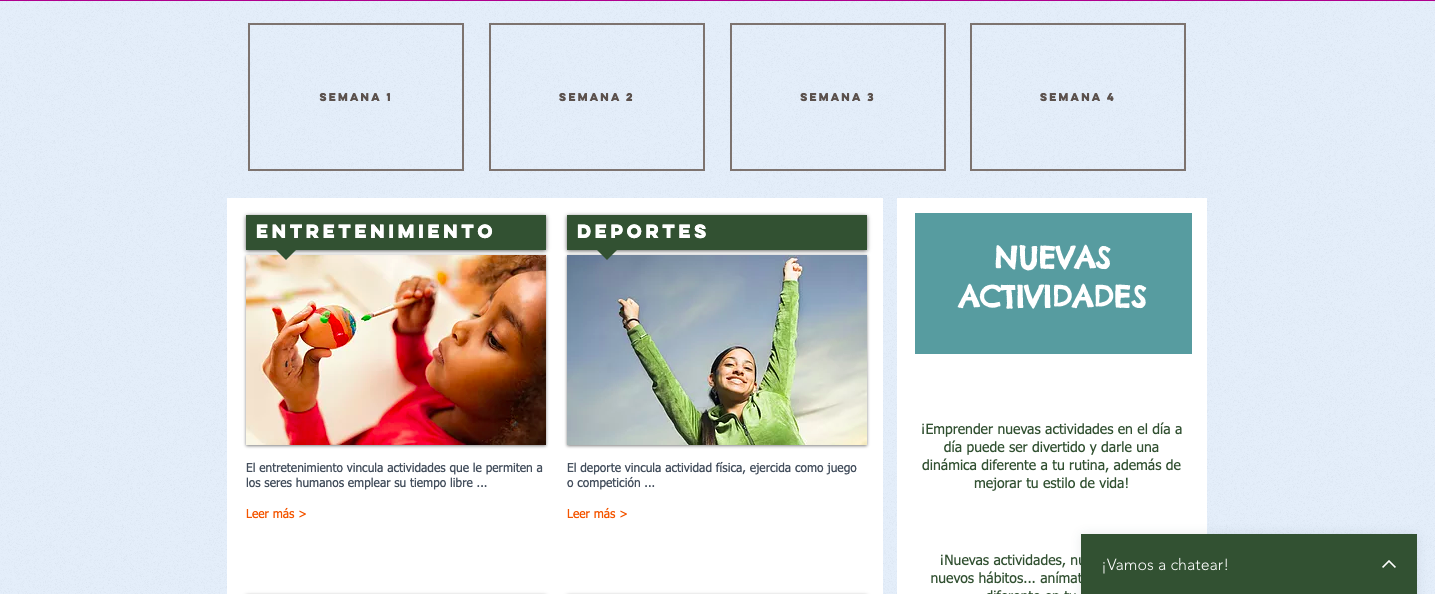


Note:

Illustrative screenshot of the “Lifestyle Diary” platform used in the study. The image shown is a royalty-free stock image included solely for demonstration purposes. No real participant is depicted.
