# Supplementary material for: Behavioral spillover between the use of reusable shopping bags and recycling at home: A field experiment
Source: PLoS One. 2025 Aug 11;20(8):e0328259. doi: 10.1371/journal.pone.0328259 (PMC12338783; doi:10.1371/journal.pone.0328259)
Supplement: S2 Appendix — (DOCX) [file pone.0328259.s002.docx]

**S2 Appendix. Regression models (no significant effects).**

**Table A. Regression Model for Recycling Behavior (Number of Bags with Recyclable Material per person; per sqm).**

| **Variables** | **Recycling (Number of bags)** | | | | | | | |
| --- | --- | --- | --- | --- | --- | --- | --- | --- |
|  | *Per person* | | | | *Per sqm* | | | |
|  | B | SE | z | p | B | SE | z | p |
| Constant/Intercept | 13.74 | 9.26 | 1.48 | .14 | .05 | .32 | .17 | .87 |
| Treatment (vs. control)  *(Use of reusable bags)* | .35 | .86 | .41 | .68 | -.04 | .04 | -1.11 | .27 |
| ***Variables of interest*** |  |  |  |  |  |  |  |  |
| Perceived Difficulty (recycling) | -.11 | .55 | -.20 | .85 | .013 | .021 | .64 | .53 |
| Self-efficacy (recycling) | .05 | .41 | .11 | .91 | .017 | .016 | 1.01 | .31 |
| General PCE | -2.40 | .85 | -2.81 | **.00** | -.10 | .031 | -3.28 | **.00** |
| Specific PCE (recycling) | 1.17 | .88 | 1.33 | .18 | .022 | .037 | .59 | .56 |
| ***Control variables*** |  |  |  |  |  |  |  |  |
| Environmental Concern | -.06 | .14 | -.42 | .68 | -.000 | .005 | -.08 | .94 |
| Moral Norm (recycling) | -.34 | .58 | -.59 | .56 | .008 | .021 | .41 | .69 |
| Social Norm (recycling) | .99 | .46 | 2.17 | **.03** | .004 | .019 | .24 | .81 |
| Environmental Identity | -1.83 | 1.32 | -1.38 | .17 | .048 | .066 | .73 | .46 |
| Knowledge (recycling) | 1.90 | 1.03 | 1.84 | .07 | .025 | .046 | .54 | .59 |
| Attitude (recycling) | -1.19 | 1.29 | -.93 | .36 | -.009 | .046 | -.19 | .85 |
| **Gender** |  |  |  |  |  |  |  |  |
| *Male* | .93 | .89 | 1.05 | .30 | -.013 | .043 | -.30 | .76 |
| Age | -.16 | .07 | -.24 | .81 | .003 | .002 | 1.28 | .20 |
| **Marital Status** |  |  |  |  |  |  |  |  |
| *Married* | -.75 | .87 | -.86 | .39 | -.075 | .031 | -2.38 | **.02** |
| *Living Together* | -1.62 | 1.39 | -1.16 | .24 | -.076 | .051 | -1.51 | .13 |
| *Divorced* | -.76 | 2.77 | -.27 | **.**79 | -.13 | .103 | -1.25 | .21 |
| **Level of Education** |  |  |  |  |  |  |  |  |
| *Undergraduate* | -.53 | 1.50 | -.35 | **.**73 | -.10 | .06 | -1.63 | .10 |
| *Graduate (post-graduate diploma)* | -2.67 | 1.01 | -2.64 | **.01** | -.10 | .04 | -2.60 | .**00** |
| *Graduate (master)* | -1.02 | 1.50 | -.68 | .50 | -.07 | .06 | -1.20 | .23 |
| **Occupation** |  |  |  |  |  |  |  |  |
| *Student* | .85 | 1.28 | .66 | .51 | -.01 | .049 | -.19 | .85 |
| *Independent job* | -.74 | 1.21 | -.61 | .54 | -.05 | .044 | -1.16 | .25 |
| *Other* | .59 | 1.52 | .39 | .70 | .09 | .058 | 1.56 | .12 |
| Income | .20 | .31 | .64 | .52 | .017 | .011 | 1.59 | .11 |
| **Type of housing** |  |  |  |  |  |  |  |  |
| *Building (apartment complex)* | 1.85 | .77 | 2.39 | **.02** | .030 | .028 | 1.06 | .29 |
| *Houses complex* | 2.14 | 1.46 | 1.47 | .15 | .042 | .052 | .81 | .42 |
| *Other* | 1.44 | 1.86 | .77 | .44 | .088 | .064 | 1.38 | .17 |
| **Household size** |  |  |  |  |  |  |  |  |
| *# People in the home* |  |  |  |  | -.018 | .011 | -1.65 | .10 |
| *Area (sqm)* | -.00 | .00 | -.70 | .48 |  |  |  |  |

**Table B. Regression Model for recycling behavior (Outside home).**

| **Variables** | **Recycling Outside Home** | | | |
| --- | --- | --- | --- | --- |
|  | B | SE | z | p |
| Constant/Intercept | 12.29 | 4.44 | 2.77 | **.00** |
| Treatment (vs. control)  *(Use of reusable bags)* | -.58 | .51 | -1.13 | .26 |
| ***Variables of interest*** |  |  |  |  |
| Perceived Difficulty (recycling) | -.03 | .29 | -.10 | .92 |
| Self-efficacy (recycling) | -.14 | .21 | -.65 | .52 |
| General PCE | -.94 | .38 | -2.47 | **.01** |
| Specific PCE (recycling) | .32 | .47 | .68 | .49 |
| ***Control variables*** |  |  |  |  |
| Environmental Concern | -.05 | .07 | -.72 | .48 |
| Moral Norm (recycling) | .85 | .26 | 3.33 | **.00** |
| Social Norm (recycling) | .11 | .23 | .46 | **.**65 |
| Environmental Identity | -.19 | .93 | -.20 | .84 |
| Knowledge (recycling) | -.17 | .63 | -.27 | .79 |
| Attitude (recycling) | -1.87 | .57 | -3.26 | **.00** |
| **Gender** |  |  |  |  |
| *Male* | -.11 | .58 | -.20 | .85 |
| Age | -.01 | .03 | -.41 | .68 |
| **Marital Status** |  |  |  |  |
| *Married* | -.03 | .39 | -.08 | .93 |
| *Living Together* | -1.62 | .64 | -2.53 | **.01** |
| *Divorced* | 1.13 | 1.38 | .81 | **.**42 |
| **Level of Education** |  |  |  |  |
| *Undergraduate* | -.60 | .89 | -.68 | **.**50 |
| *Graduate (specialization)* | -.20 | .47 | -.43 | .67 |
| *Graduate (master)* | -.21 | .77 | -.27 | .79 |
| **Occupation** |  |  |  |  |
| *Student* | .70 | .68 | 1.02 | .31 |
| *Independent job* | -.52 | .54 | -.10 | .33 |
| *Other* | .88 | .83 | 1.06 | .29 |
| Income | .14 | .14 | 1.02 | .31 |
| **Type of housing** |  |  |  |  |
| *Building (apartment complex)* | .64 | .35 | 1.81 | .07 |
| *Houses complex* | .86 | .65 | 1.31 | .19 |
| *Other* | 1.14 | .90 | 1.27 | .21 |
| **Household size** |  |  |  |  |
| *# People in the home* | -.09 | .18 | -.50 | .62 |
| *Area (sqm)* | .00 | .00 | .33 | .74 |

Note: reference category: Gender (Female); Marital Status (Single); Level of Education (High School); Occupation (Employee); Type of housing (Single-family home)

Source: Author’s own elaboration
