# Supplementary material for: Behavioral spillover between the use of reusable shopping bags and recycling at home: A field experiment
Source: PLoS One. 2025 Aug 11;20(8):e0328259. doi: 10.1371/journal.pone.0328259 (PMC12338783; doi:10.1371/journal.pone.0328259)
Supplement: S3 Appendix — (DOCX) [file pone.0328259.s003.docx]

**S3 Appendix. GEE panel data regressions of recycling at home, per person, with interaction terms.**

|  | **Coeff.** | **S.E** | **z** | **p-value** | **95% CI** | |
| --- | --- | --- | --- | --- | --- | --- |
| Treatment (1 = intervention) | 4.417 | 0.913 | 4.840 | 0.000 | 2.628 | 6.205 |
| EDAD | -0.016 | 0.011 | -1.420 | 0.157 | -0.038 | 0.006 |
| Married* | 0.236 | 0.133 | 1.770 | 0.077 | -0.026 | 0.498 |
| Living Together | 0.661 | 0.364 | 1.810 | 0.070 | -0.053 | 1.375 |
| Divorced | 0.877 | 0.837 | 1.050 | 0.295 | -0.763 | 2.517 |
| Undergraduate* | 1.506 | 0.454 | 3.310 | 0.001 | 0.615 | 2.397 |
| Graduate (post-graduate) | 0.852 | 0.262 | 3.250 | 0.001 | 0.338 | 1.367 |
| Graduate (master) | 1.077 | 0.380 | 2.830 | 0.005 | 0.332 | 1.822 |
| Gander (male)* | 0.195 | 0.133 | 1.460 | 0.143 | -0.066 | 0.456 |
| Attitude to recycling | -0.425 | 0.253 | -1.680 | 0.094 | -0.921 | 0.072 |
| Environmental Knowldege | 0.316 | 0.101 | 3.140 | 0.002 | 0.119 | 0.514 |
| Identity with recycling | -0.474 | 0.176 | -2.690 | 0.007 | -0.819 | -0.129 |
| Social norms (recycling) | 0.228 | 0.101 | 2.250 | 0.025 | 0.029 | 0.426 |
| Moral norms (recycling) | 0.097 | 0.211 | 0.460 | 0.646 | -0.316 | 0.510 |
| Environmemtal concern | 0.011 | 0.037 | 0.300 | 0.766 | -0.062 | 0.084 |
| Perceived Difficulty (recycling) | 0.334 | 0.068 | 4.940 | 0.000 | 0.202 | 0.466 |
| Self-efficacy (recycling) | 0.263 | 0.086 | 3.050 | 0.002 | 0.094 | 0.433 |
| General PCE | -0.365 | 0.191 | -1.910 | 0.056 | -0.740 | 0.009 |
| Specific PCE (recycling) | 0.325 | 0.142 | 2.280 | 0.023 | 0.046 | 0.604 |
| **Interaction effects** |  |  |  |  |  |  |
| Treatment*Perceived difficulty | -0.220 | 0.111 | -1.970 | 0.048 | -0.438 | -0.002 |
| Treatment*Self efficacy | -0.318 | 0.123 | -2.580 | 0.010 | -0.560 | -0.077 |
| Treatment*PCE | -0.372 | 0.173 | -2.150 | 0.032 | -0.711 | -0.032 |
| _cons | -1.660 | 1.239 | -1.340 | 0.180 | -4.088 | 0.769 |

*Note: These categorical variables were dummy coded with the following reference category: Gender (Female); Marital Status (Single); Level of Education (High School)
